# Fetal renal cystic disease and post-natal follow up—a single center experience

**DOI:** 10.3389/fped.2023.1243504

**Published:** 2023-08-10

**Authors:** Lorena Botero-Calderon, Anne Lawrence, Natalie O’Toole, Lisa M. Guay-Woodford

**Affiliations:** ^1^ Division of Nephrology, Children’s National Hospital, Washington, DC, United States; ^2^ Prenatal Pediatrics Institute, Children’s National Hospital, Washington, DC, United States; ^3^ Center for Translational Research, Children’s National Research Institute, Washington, DC, United States

**Keywords:** echogenic fetal kidneys, transition planning, renal ciliopathies, hepato-renal fibrocystic diseases, polycystic kidney diseases

## Abstract

**Introduction:**

Prenatal sonographic evidence of large, echogenic, or cystic kidneys may indicate any one of a diverse set of disorders including renal ciliopathies, congenital anomalies of the kidney and urinary tract (CAKUT), or multisystem syndromic disorders. Systematic transition planning for these infants from *in utero* detection to post-natal nephrology management remains to be established.

**Aim of the work:**

We sought to evaluate the presentation and transition planning for infants identified *in utero* with bilateral renal cystic disease.

**Methods:**

Our retrospective observational study identified 72 pregnancies with bilateral renal cystic disease in a single center from 2012 to 2022; 13 of which had a confirmed renal ciliopathy disorder. Clinical and imaging data, genetic test results, and documentation of postnatal follow-up were collected and compared.

**Results:**

In our suspected renal ciliopathy cohort (*n* = 17), autosomal recessive polycystic disease (ARPKD) was the most common diagnosis (*n* = 4), followed by Bardet-Biedl syndrome (BBS, *n* = 3), autosomal dominant polycystic disease (ADPKD, *n* = 2), *HNF1B*-related disease (*n* = 2), and Meckel-Gruber syndrome (MKS, *n* = 2). Four cases were not genetically resolved. Anhydramnios was observed primarily in fetuses with ARPKD (*n* = 3). Polydactyly (*n* = 3) was detected only in patients with BBS and MKS, cardiac defects (*n* = 6) were identified in fetuses with ARPKD (*n* = 3), MKS (*n* = 2), and BBS (*n* = 1), and abnormalities of the CNS (*n* = 5) were observed in patients with ARPKD (*n* = 1), MKS (*n* = 2), and BBS (*n* = 3). In general, documentation of transition planning was incomplete, with post-natal nephrology management plans established primarily for infants with renal ciliopathies (*n* = 11/13; 85%).

**Conclusion:**

Prenatal sonographic detection of echogenic kidneys should raise suspicion for a broad range of disorders, including renal ciliopathies and CAKUT. Multicenter collaboration will be required to standardize the implementation of transition guidelines for comprehensive nephrology management of infants identified *in utero* with enlarged, echogenic kidneys.

## 1. Introduction

Renal anomalies are detected in 10%–20% of prenatal ultrasounds (1). These anomalies are most commonly described as bilateral enlarged and/or hyperechogenic kidneys and can be clustered into three major groups. The first comprises the inherited hepatorenal fibrocystic diseases (HRFD), a set of monogenic disorders characterized by fibrocystic abnormalities of the kidney and dysgenesis of the porto-biliary tract. These disorders involve mutations in genes encoding proteins that function in the primary cilium or centrosome, and thus HRFDs are considered a renal subset of the larger group of ciliopathy disorders (2). In childhood, the polycystic kidney diseases, e.g., autosomal recessive polycystic kidney disease (ARPKD) and autosomal dominant polycystic kidney disease (ADPKD), are the most common renal ciliopathies or HRFDs, followed by nephronophthisis (NPHP), Joubert syndrome (JBTS), Meckel-Gruber syndrome (MKS), and Bardet-Biedl syndrome (BBS) (2). The second group encompasses the congenital anomalies of the kidney and urinary tract (CAKUT) spectrum of disorders, which can present as renal hypoplasia, ureteropelvic junction obstruction (UPJO), primary megaureter, vesicoureteral reflux (VUR), ureterovesical junction obstruction (UVJO), or posterior urethral valves (PUV), with or without associated renal cystic disease (3, 4). The final group involves patients with chromosomal or syndromic disorders. Clinical presentation in all groups can vary from mild to severe with anhydramnios and pulmonary hypoplasia causing significant perinatal mortality (5). From a sonographic imaging perspective, these diverse set of disorders often mimic or phenocopy one another.

Taken as a whole, renal cystic diseases are a common cause of pediatric end-stage kidney disease (ESKD), with an overall incidence of approximately 4 cases per 10,000 births (6). Among the HRFDs, ADPKD can present in the perinatal period, over the course of childhood, or in young adulthood, and is caused primarily by mutations in two genes, *PKD1* (78% of cases) and *PKD2* (15% of cases), with the remaining 5%–10% of cases due to rare mutations in other loci (7). In comparison, ARPKD is less frequent, with an incidence of 1 in 26,500 live births (8). It is caused primarily by mutations in the *PKHD1* gene (9). In its most severe antenatal presentation, ARPKD is characterized by bilaterally enlarged echogenic kidneys and oligohydramnios, with 21% perinatal mortality due to pulmonary hypoplasia (8, 10). Surviving patients typically reach adulthood, due to medical advances in childhood kidney replacement therapy (KRT) and kidney transplantation (11). The rarer forms of HRFDs, such as NPHP, JBTS, MKS, and BBS, are characterized not only by renal cystic disease, but can involve diverse extra-renal features including polydactyly, occipital encephalocele, hepatic fibrosis, obesity, and retinal degeneration.

CAKUT represents a diverse group of structural malformations originating from a failure at any point during the embryonic development of the kidney and urinary tract. It is the main cause of ESKD in childhood (∼40%) with an incidence of 3–6 affected children per 1,000 births (12). Clinical presentation can vary from isolated renal anomalies to syndromic phenotypes. Familial clustering has been observed. More than 50 genes have been implicated in CAKUT. The most common mutations involve 12 genes, e.g., *BMP7*, *CDC5l*, *CHD1l*, *EYA1*, *GATA3*, *HNF1B*, *PAX2*, *RET*, *ROBO2*, *SALL1*, *SIX2*, and *SIX5* (13). However, single-gene mutations only explain 10% to 20% of the CAKUT cases (14). In addition, copy number variants (CNVs), i.e., duplications or deletions of genomic segments, underlie 5%–10% of CAKUT cases. A recent high-density SNP array analysis involving almost 3,000 patients with CAKUT and more than 21,000 control individuals identified numerous disease-associated CNVs (45 at 37 loci), consistent with the known genetic heterogeneity of CAKUT. Notably, in the majority of patients (65%) CNVs were detected in 6 loci (1q21, 4p16.1–p16.3, 16p11.2, 16p11.3, 17q12, 22q11.2), suggesting that these are critical regions in the development of kidney and urinary tract (15).

In this retrospective study, we reviewed 11-years of data from our Prenatal Pediatrics Institute program and identified all cases of prenatally detected echogenic and/or enlarged kidney with or without cysts. We compiled the clinical and imaging data with available genetic testing results and post-natal management plans. This single center study demonstrates that for infants with bilateral renal cystic disease there is often a gap in transition planning from prenatal diagnosis to postnatal specialty management.

## 2. Methods and materials

This was a retrospective observational study for an 11-year period from January of 2012 to December of 2022. Pregnant mothers were referred to the Prenatal Pediatrics Institute by their primary providers for evaluation of oligo- or anhydramnios, and/or urogenital, cardiac, or CNS malformations on screening sonography. Evaluation included detailed obstetric ultrasound as well as fetal echocardiography and fetal MRI with fetal brain MRI, when indicated.

We reviewed the Prenatal Pediatrics Institute databases to identify fetuses diagnosed with genito-urinary (GU) tract abnormalities at the initial evaluation and then used the search terms of polycystic kidney disease, renal cyst, enlarged kidney, and echogenic kidney to identify a subset of fetuses with various forms of kidney cysts (*n* = 105). Spot review of individual patient charts revealed that a number of patients had both renal cystic disease and GU tract anomalies. Therefore, we conducted a further detailed review using the inclusion criteria of enlarged and/or echogenic kidneys with or without cysts to capture the subset of patients with bilateral renal cystic disease as their predominant anomaly (*n* = 72).

For each of these 72 fetuses and their mothers, clinical, imaging, and genetic information was extracted from the Prenatal Pediatrics Institute databases, as well as the maternal medical records, and captured in a structured REDCap-based Database. The data elements included: demographics (sex and race), maternal information (age, history of diabetes and/or obesity), imaging findings (enlarged/echogenic kidneys with or without cystic changes, cysts size, major extrarenal anomalies including cardiac, skeletal, gastrointestinal, and CNS), perinatal outcomes of affected fetuses [survival rate, admission to the neonatal intensive care unit (NICU), need for ventilatory support], genetic evaluation platforms (karyotype, FISH, microarray, personalized sequencing panel, exome sequencing) and nephrology follow up. The data were analyzed using descriptive statistics. The Institutional Board Review (IRB) of Children's National Hospital approved the study (ID: Pro00016398).

## 3. Results

### 3.1. Referrals to the Perinatal Pediatrics Institute

Our analyses determined that on average there were 600–700 referrals to the Prenatal Pediatrics Institute per year between 2012 and 2022 (Table 1). With the implementation of telemedicine during the COVID-19 pandemic, there was a significant increase in referrals to 981 in 2022. Year over year, 80%–90% of the referrals had at least one type of fetal anomaly. However, anomalies of the genitourinary (GU) tract comprised only 7%–22% of the referral cases.

**Table 1:** Referrals to the prenatal pediatric institute.

Year	TOTAL Referrals	TOTAL Abnormal (%)	TOTAL GU tract abnormalities (%)	TOTAL GU anomalies^1^ (%)	TOTAL Kidney cysts^2^ (%)	TOTAL Renal cystic disease^3^ (%)
2012	570	515 (90%)	34 (7%)	27 (79%)	7 (21%)	5 (71%)
2013	614	522 (85%)	96 (18%)	91 (95%)	5 (5%)	4 (80%)
2014	623	546 (88%)	107 (19%)	101 (94%)	6 (6%)	3 (50%)
2015	623	557 (89%)	95 (17%)	88 (93%)	7 (7%)	3 (43%)
2016	727	650 (89%)	120 (18%)	108 (90%)	12 (10%)	8 (67%)
2017	763	672 (88%)	118 (17%)	106 (90%)	12 (10%)	10 (83%)
2018	704	606 (86%)	118 (19%)	109 (92%)	9 (8%)	6 (67%)
2019	668	565 (85%)	92 (16%)	86 (93%)	6 (7%)	4 (67%)
2020	672	598 (89%)	136 (22%)	121 (89%)	15 (11%)	11 (73%)
2021	792	710 (90%)	111 (15%)	101 (91%)	10 (9%)	8 (80%)
2022	981	899 (92%)	132 (15%)	116 (88%)	16 (12%)	10 (63%)
**TOTAL**	**7,737**	**6,840** (88%)	**1,159** (**17%)**	**1,054** (**91%)**	**105** (**9%)**	**72** (69%)

^1^ TOTAL number of cases with GU anomalies: duplex collecting system, dysplastic kidney, hydronephrosis, hydroureter, MCDK, pyelectasis, PUV, UPJ.

^2^ TOTAL number of cases with Cystic kidneys: cystic kidney, echogenic kidney, enlarged kidney, polycystic kidney, renal cyst.

^3^ TOTAL number of cases with Renal cystic disease: subset with echogenic and/or enlarged kidney with or without cysts.

Step-wise analysis of this cohort revealed that 5%–21% had some form of kidney cysts whereas 79%–95% primarily had other, extra-renal GU anomalies. We further filtered the cases with renal cysts to enrich for patients with predominantly bilateral renal cystic disease (*n* = 72), eliminating patients with both cystic kidneys and GU tract anomalies where CAKUT was the likely diagnosis of the cystic kidney.

### 3.2. Maternal characteristics

Mothers of this bilateral renal cystic disease cohort primarily self-identified as Caucasians (29%) or African-Americans (29%), with Hispanics (14%) and Asians (11%) each comprising substantial fractions of the cohort (Table 2). While African-Americans were somewhat over-represented in this cohort, the racial and ethnic distribution generally corresponds to the demographics of metropolitan Washington DC referral base (16).

**Table 2:** Maternal-fetal characteristics for patients with renal cystic disease.

	(*N*)	
Median maternal age	72	35.5 years
	(*N*)	Percentage (%)
Self-identified race (*n* = 72)		
Caucasian	21	29
African-American	21	29
Asian	8	11
Hispanic	10	14
Unknown	12	17
Maternal obesity (*n* = 72)		
Yes	25	35
No	25	35
Unknown	22	30
Maternal diabetes (*n* = 72)		
Yes	11	15
No	40	56
Unknown	21	29
Fetal sex (*n* = 72)		
Male	39	54
Female	28	39
Unknown	5	7
Infant born alive (*n* = 72)		
Yes	52	72
Termination of pregnancy (TOP)	14	19
Stillborn	1	2
Unknown	5	7
Ventilatory support among NICU admissions (*n* = 41)		
Yes	16	39
No	25	61
Neonatal survival (*n* = 72)		
Alive	24	33
Deceased/TOP	32	45
Unknown	16	22

The mean maternal age of the renal cystic disease cohort was 35.5 years (Table 2). Maternal obesity was documented in 25 (35%) pregnancies. However, there was no specific documentation in an additional 22 (30%) pregnancies, suggesting that this clinical feature was not consistently captured, Interestingly, maternal diabetes was documented in 11 (15%) of all pregnancies affected, but only present in 4/13 mothers with fetuses with genetically-confirmed renal ciliopathies.

### 3.3. Patient characteristics

The renal cystic disease cohort (*n* = 72) was predominantly male (54%, sex unknown 7%) (Table 2).

Perinatal and postnatal outcomes were assessed. The mean gestational age (GA) at birth was 40 weeks. Perinatal survival was high (90%) when termination of pregnancy (TOP) was excluded. For the 14 (19%) TOP, families cited poor prognosis due to multiple congenital malformations as the most common rationale. There was only one stillbirth and no known spontaneous pregnancy losses. Among the 52 infants born alive, admission to the neonatal intensive care unit (NICU) was common, but not universal. Somewhat surprisingly, only 39% of these neonates required ventilatory support. Using an active medical record as a proxy for post-natal survival, we could document survival beyond the neonatal period in 33% of infants. However, the status was unknown for approximately a quarter of the cohort, which were presumably lost to follow-up.

### 3.4. Clinical characteristics of cohort with bilateral renal cystic disease

Our sequential analyses identified 72 patients that met our criteria for bilateral renal cystic disease (Table 3). With further detailed review of individual medical records, we identified 17 (24%) cases of suspected renal ciliopathies. Among the remaining cases, we identified renal cystic disease associated with 1) urinary tract anomalies, i.e., CAKUT, (*n* = 10; 14%); 2) chromosomal abnormalities (*n* = 6; 8%), and 3) syndromic disorders (*n* = 9; 12%). Another 6 (8%) infants had enlarged, echogenic kidneys at birth, but were subsequently lost to follow-up. Thirteen (18%) pregnancies ended in either TOP or stillbirth; for 8 (11%) live-born infants there was no post-natal evaluation documented in the medical record. In these cases, the renal cystic disease was not further characterized. Finally, for 3 (4%) neonates, post-natal evaluation revealed structurally normal kidneys.

**Table 3:** Clinical characteristics of cohort with renal cystic disease (*N* = 72).

Diagnostic category	Sex	Race	Amniotic fluid^1^	Systemic anomalies	Follow up
Suspected ciliopathy (*n* = 17)	Male (*n* = 11) Female (*n* = 6)	Black (*n* = 6) White (*n* = 5) Hispanic (*n* = 3) Asian (*n* = 1) Unknown (*n* = 2)	Normal (*n* = 11) Polyhydramnios (*n* = 1) Oligohydramnios (*n* = 2) Anhydramnios (*n* = 3)	CNS (*n* = 6) Cardiac (*n* = 6) GI (*n* = 2) Skeletal (*n* = 3)	Inherited and Polycystic Kidney Diseases Program (*n* = 8) General Nephrology (*n* = 3) No Nephrology follow up (*n* = 2) Perinatal demise (*n* = 4)
CAKUT (*n* = 10)	Male (*n* = 5) Female (*n* = 5)	Black (*n* = 1) White (*n* = 4) Hispanic (*n* = 3) Asian (*n* = 1) Unknown (*n* = 1)	Normal (*n* = 6) Oligohydramnios (*n* = 4)	CNS (*n* = 3) Cardiac (*n* = 1) GI (*n* = 2) Skeletal (*n* = 1)	Inherited and Polycystic Kidney Diseases Program (*n* = 2) General Nephrology (*n* = 1) No Nephrology follow up (*n* = 3) Perinatal demise (*n* = 4)
Chromosomal abnormalities (*n* = 6) - Trisomy 13 - Monosomy X - Large interstitial deletion of chromosome 9q22.2q22.32	Male (*n* = 2) Female (*n* = 3) Not defined (*n* = 1)	White (*n* = 1) Hispanic (*n* = 2) Asian (*n* = 1) Unknown (*n* = 2)	Normal (*n* = 4) Oligohydramnios (*n* = 2)	CNS (*n* = 6) Cardiac (*n* = 5) GI (*n* = 4) Skeletal (*n* = 3)	General Nephrology (*n* = 1) No Nephrology follow up (*n* = 2) Perinatal demise (*n* = 3)
Syndromes (*n* = 9) - Lethal neonatal CPTII deficiency - Denys-Drash - Congenital hydrocephalus s/p VP shunt, epilepsy - Gastroschisis - Beckwith-Wiedemann - Macrocephaly capillary malformation and polymicrogyria syndrome s/p VP shunt - Heterozygous for POMT2 gene associated with muscular dystrophy - Congenital intracranial cysts	Male (*n* = 5) Female (*n* = 4)	Black (*n* = 4) White (*n* = 1) Asian (*n* = 2) Unknown (*n* = 2)	Normal (*n* = 4) Polyhydramnios (*n* = 2) Oligohydramnios (*n* = 3)	CNS (*n* = 6) Cardiac (*n* = 1) GI (*n* = 2) Skeletal (*n* = 2)	General Nephrology (*n* = 1) No Nephrology follow up (*n* = 5) Perinatal demise (*n* = 3)
Echogenic/enlarged kidneys at birth (*n* = 6)	Male (*n* = 3) Female (*n* = 2) Not defined (*n* = 1)	Black (*n* = 4) White (*n* = 1) Unknown (*n* = 1)	Normal (*n* = 5) Oligohydramnios (*n* = 1)	CNS (*n* = 1)	No Nephrology follow up (*n* = 4) General Nephrology (*n* = 2)
TOP or stillborn (*n* = 13)	Male (*n* = 5) Female (*n* = 5) Not defined (*n* = 3)	Black (*n* = 4) White (*n* = 5) Asian (*n* = 3) Unknown (*n* = 1)	Normal (*n* = 10) Anhydramnios (*n* = 3)	CNS (*n* = 7) Cardiac (*n* = 8) GI (*n* = 5) Skeletal (*n* = 5)	** **
Undiagnosed/lost to follow-up (*n* = 8)	Male (*n* = 4) Female (*n* = 3) Not defined (*n* = 1)	Black (*n* = 1) Hispanic (*n* = 2) White (*n* = 2) Unknown (*n* = 3)	Normal (*n* = 3) Polyhydramnios (*n* = 2) Oligohydramnios (*n* = 2) Anhydramnios (*n* = 1)	CNS (*n* = 3) Cardiac (*n* = 2) GI (*n* = 1) Skeletal (*n* = 3)	No Nephrology follow up (*n* = 5) Perinatal demise (*n* = 3)
Post-natal normal kidneys (*n* = 3)	Male (*n* = 3)	Black (*n* = 1) White (*n* = 2)	Normal (*n* = 3)		Inherited and Polycystic Kidney Diseases Program (*n* = 1) No Nephrology follow up (*n* = 2)

^1^ Polyhydramnios, oligohydramnios, and anhydramnios were characterized based on the amniotic fluid index (AFI), with polyhydramnios defined as AFI ≥20 cm, oligohydramnios as an AFI of ≤5 cm, and anhydramnios as complete absence of amniotic fluid.

For this study, we defined polyhydramnios as amniotic fluid index (AFI) ≥20 cm, oligohydramnios as an AFI of ≤5 cm, and anhydramnios as complete absence of amniotic fluid. Fetuses with suspected renal ciliopathies and TOP/stillborn had the highest incidence of anhydramnios, with 3 cases in each group. There were 5 cases with polyhydramnios, one in the suspected ciliopathies cohort, two in the syndromes group and two in the undiagnosed/lost to follow up group, respectively.

The most frequent systemic anomalies affected the central nervous system (CNS) (*n* = 33/72, 46%), followed by cardiac anomalies (*n* = 22/72, 31%), and skeletal anomalies (*n* = 17/72, 24%) (Table 3).

We were specifically interested in the post-natal referral of these patients for nephrology follow-up, e.g., at least one visit to either the general nephrology clinic or the Children's National Hospital (CNH) Inherited Renal Disorders (IRD) Program as documented in the electronic medical record (EMR). Only 11 infants (15%) were evaluated in the IRD Program and 6 (8%) cases in the general nephrology clinic. Among the subset of patients with genetically confirmed ciliopathies, 11/13 (54%) had nephrology follow up, with 8 cases being seen in the IRD clinic and 3 cases in the general nephrology clinic. The remaining infants did not have longitudinal nephrology follow up according to EMR documentation (Table 3).

### 3.5. Genetic findings

In our bilateral renal cystic disease cohort, 13/72 fetuses (18%) had genetic confirmation of a renal ciliopathy disorder (Table 4). There was no history of consanguinity for any of these pregnancies. All cases of ADPKD had a positive family history in accordance with the autosomal dominant inheritance pattern. The primary genetic diagnostic platform was a customized next generation sequencing panel (CNH Personalized Sequencing Panel). Other methods included targeted *PKHD1* gene sequencing, sequence analysis and deletion/duplication testing, and slice multi-gene testing. Only one fetus with ARPKD underwent exome sequencing.

**Table 4:** Genetic characterization in fetuses with prenatally suspected ciliopathies (*n *= 17).

Diagnosis	Sex	Consanguinity	Family history	Testing platform	Genetic results
ARPKD (*n *= 4)	Male (*n *= 3) Female (*n *= 1)	No/unknown (*n *= 4)	No (*n *= 4)	Ciliopathies panel (*n *= 1)	*PKHD1* c.107C>T; p.Thr36Met (pathogenic moderate; ACMG Classification) *PKHD1* c.8302+5G>A (pathogenic splice variant)
				Direct mutation analysis (*n *= 1)	*PKHD1* c.3761_3762delCCinsG; p.Ala1254fs *PKHD1* c.9370C>T p.His3124Tyr (pathogenic moderate; ACMG Classification)
				Personalized sequencing panel (*n *= 1)	Homozygous *PKHD1* c.7497delT; p.Val2500fs
				Whole exome sequencing (*n *= 1)	*PKHD1* c.930delC; p.Thr311fs *PKHD1* c.619 G>T; p.Asp207Tyr (VUS; ACMG classification)
ADPKD (*n *= 2)	Male (*n *= 2)	No (*n *= 2)	Yes (*n *= 2)	Not done; + family hx (*n *= 1)	—
				Personalized sequencing panel (*n *= 1)	*PKD1* c.9499A>T; p.Ile3167Phe (benign; ACMG classification)
*HNF1b*-related renal cystic disease (*n *= 2)	Male (*n *= 2)	No	No	Personalized sequencing panel	*HNF1b* c.826C>T; p.Arg276*
					*HNF1b* c.544+1G>A; Intron 2
Meckel-Gruber syndrome (*n *= 2)	Female (*n *= 2)	No	No	Ciliopathies panel (*n *= 1)	*CEP290* c.2722C>T; p.Arg908* CEP290 c.5012+2T>C (pathogenic splice variant)
				Joubert and Meckel-Gruber syndrome next generation sequencing panel (*n *= 1)	*RPGRIP1L* c.149delT; p.Leu50fs *RPGRIP1L* c.1709dupA; Asp571fs
Bardet-Biedl syndrome (*n *= 3)	Male (*n *= 3)	No	No	Personalized sequencing panel (*n *= 2)	Heterozygous *BBS10* c.1000_1001insGA; p.Leu334fs Homozygous *BBS10* c.2119_2120delGT; p.Val707fs
					Homozygous *BBS10* c.1677delC; p.Tyr559fs
				Next generation sequencing (*n *= 1)	Homozygous *BBS2* c.1225+2dup (pathogenic splice variant)
Genetically unresolved suspected renal ciliopathies (*n *= 4)	Male (*n *= 1) Female (*n *= 3)	No/unknown (*n *= 4)	No (*n *= 4)	Personalized sequencing panel (*n *= 1)	Heterozygous *PKD2* c.1904C; p.T6351 (VUS; ACMG classification)

### 3.6. Ultrasound findings and outcomes of fetuses with confirmed renal ciliopathies

The suspected renal ciliopathy cohort was evenly distributed between fetuses diagnosed with polycystic kidney disease (*n* = 6/13, 46%) and syndromic ciliopathies (*n* = 7/13, 54%) (Table 5). Amongst the PKD cases, ARPKD (*n* = 4/6, 67%) was the more frequent disorder compared to ADPKD (*n* = 2/6, 33%). The group of syndromic ciliopathies consisted of BBS (*n* = 3/7, 43%), MKS (*n* = 2/7, 29%), and *HNF1B*-related renal cystic disease (*n* = 2/7, 29%). The cohort was predominantly male (77%).

**Table 5:** Ultrasound characteristics of fetuses with prenatally suspected renal ciliopathies (*n* = 17).

Diagnosis	Sex	Amniotic fluid index	Kidney	Liver	Skeletal anomalies	CNS abnormalities	Cardiac defects	Outcome
ADPKD	M	Normal	Echogenic, enlarged	Normal	None	None	None	Live born
	M	Normal	Echogenic, enlarged	Not assessed	None	None	None	Live born
ARPKD	M	Anhydramnios	Cystic, echogenic, enlarged	Normal	None	None	Pericardial effusion	Perinatal demise
	M	Anhydramnios	Cystic, echogenic, enlarged	Normal	None	None	Pericardial effusion	Perinatal demise
	M	Anhydramnios	Cystic, echogenic, enlarged	Normal	None	Ventriculomegaly	Thickened myocardium	Perinatal demise
	F	Oligohydramnios	Cystic, echogenic, enlarged	Normal	None	None	None	Live born
*HNF1b*-related renal cystic disease	M	Normal	Cystic, echogenic	Not assessed	None	None	None	Live born
	M	Polyhydramnios	Cystic, echogenic, enlarged	Not assessed	None	None	None	Live born
Meckel-Gruber Syndrome	F	Oligohydramnios	Cystic, enlarged	Not assessed	Polydactyly, bilateral club foot	Hydrocephalus, hypoplastic vermis	HLHS^1^	Perinatal demise
	F	Normal	Cystic, enlarged	Not assessed	None	Dandy-Walker malformation, polymicrogyria, occipital bone defect	Echogenic left ventricular focus	Termination of pregnancy
Bardet-Biedl Syndrome	M	Normal	Cystic, echogenic, enlarged	Not assessed	Polydactyly	None	HLHS^1^	Live born
	M	Normal	Echogenic, enlarged	Not assessed	None	None	None	Live born
	M	Normal	Echogenic, enlarged	Not assessed	Polydactyly, club foot	Ventriculomegaly, megacisterna magna	None	Live born
Unconfirmed ciliopathies	M		Echogenic, enlarged	Not assessed		Ventriculomegaly		Unknown
	F		Cystic, echogenic	Not assessed				Unknown
	F		Cystic, echogenic, enlarged	Not assessed				Unknown
	F		Normal	Not assessed		Dandy-Walker malformation, brainstem dysplasia, cerebellar hypoplasia		Live born

^1^ HLS, hypoplastic left heart syndrome.

Hyperechoic kidneys were present in 100% (*n* = 13) of the fetuses with renal ciliopathies (Table 5). Echogenicity being defined as the degree of sonographic intensity of the kidney parenchyma in comparison to the liver. Almost all fetuses with ARPKD (*n* = 3/4, 75%) had bilateral massively enlarged kidneys; half of them had microcysts scattered throughout the renal parenchyma. Similarly, fetuses with MKS had also multiple microcysts or parenchyma replaced with innumerable small cystic structures. The kidneys of fetuses affected by BBS and ADPKD were enlarged and without evident cysts. The renal phenotype in *HNF1B-*related renal cystic disease cases were variable, characterized by loss of corticomedullary differentiation and presence of scattered cysts. Oligohydramnios or anhydramnios occurred in fetuses with ARPKD and one fetus with MKS, while there was one case of polyhydramnios in a fetus with *HNF1B-*related renal cystic disease.

While the ductal plate malformation (DPM) is an invariant histopathological finding in patients with ARPKD and can be observed in a subset of patients with early onset ADPKD, sonographic imaging abnormalities associated with the DPM are typically not evident until after birth (17). Consistent with previous observations, no imaging abnormalities of the liver were evident in the four patients with genetically resolved ARPKD and the two patients with ADPKD (Table 5).

Fetuses with MKS presented with either the classic occipital encephalocele or a Dandy-Walker malformation. Other findings included hypoplastic cerebellar vermis and polymicrogyria. Ventriculomegaly was noted in one case of ARPKD and one case of BBS, respectively. Skeletal findings such as polydactyly and club foot were seen in the MKS and BBS patients, as expected for these syndromic disorders. Cardiac abnormalities ranged from pericardial effusion in 50% (*n* = 2/4) of ARPKD cases to severe anatomical defects such hypoplastic left heart syndrome (HLHS) in one case of MKS and one case of BBS, respectively (Table 5).

With respect to perinatal outcomes, 62% (*n* = 8/13) were live born, in one patient with MKS the pregnancy was terminated, and perinatal demise occurred in 4 cases (ARPKD *n* = 3, MKS *n* = 1) (Table 5).

## 4. Discussion

Over the 11-years covered in this retrospective observational study, our Prenatal Pediatric Institute received >7,500 pregnancy referrals. As would be expected for a referral center, a fetal anomaly was detected in the overwhelming majority of cases (88%). On average, GU tract abnormalities were identified in 17% of these fetuses, with the majority (91%) having non-renal GU anomalies and only 105 (9%) having some form of kidney cysts. Among this later subset, 72/105 (69%) had bilateral renal cystic disease.

Detailed chart review revealed that only 17/72 (24%) fetuses had a suspected renal ciliopathy, with genetic resolution in 13 (76%). Among these 13 patients, the most frequent diagnosis was ARPKD (30%), followed by ADPKD and *HNF1B*-related disease, both well-known ARPKD phenocopy disorders (18). Other diagnoses included Meckel-Gruber (MKS) and Bardet-Biedl (BBS) syndrome, both multi-system ciliopathies.

ARPKD is the prototype of the hepato-renal fibrocystic diseases (19) with mutations in the *PKHD1* gene accounting for >98% of cases (20). A recent electronic health record-based analysis estimated that 21% of affected infants died in the immediate perinatal period due to the consequences of their associated pulmonary hypoplasia (8). In the current study, all 4 infants with ARPKD had the classic sonographic features of enlarged, hyperechoic kidneys. Of note, all the three that suffered perinatal demise had anhydramnios and cardiac abnormalities, e.g., pericardial effusion (*n* = 2); thickened myocardium (*n* = 1), raising the question of whether cardiac dysfunction contributed to their demise. While the association of ARPKD and cardiac impairment is not well described, a recent single center study demonstrated that children with ARPKD have significant prevalence of subclinical left ventricular hypertrophy, abnormal cardiac remodeling, and systolic mechano-dysfunction (21). However, this study was conducted in post-natal survivors across a broad age range (0–18 years). It remains to be determined whether subclinical cardiac dysfunction is prevalent in fetuses with ARPKD and whether it is an under-appreciated contributor to perinatal demise.

Among neonates presenting with enlarged, echogenic kidneys, mutations in the ADPKD genes, *PKD1* and less frequent *PKD2*, are detected almost as frequently as *PKHD1* mutations. These mutations may be vertically transmitted, arise *de novo*, or less commonly, affect both alleles in a recessive mode (22). This genetic epidemiology is consistent with our ADPKD cohort where single allele *PKD1* mutations were identified in both cases. These infants had enlarged, echogenic kidneys without gross cysts and normal amniotic fluid indices.

Mutations in *HNF1B* cause a pleotropic set of GU anomalies, including CAKUT and various forms of renal cystic disease (e.g., hyperechogenic kidneys (phenocopying ARPKD), multicystic kidney disease, renal agenesis, renal hypoplasia, cystic dysplasia, and hyperuricemic tubulointerstitial nephropathy (23–25). In our cohort, both fetuses had enlarged echogenic kidneys and one had polyhydramnios, an association that has been reported previously (26).

MKS is an autosomal recessive multisystem ciliopathy, that is usually lethal in the neonatal period. Most patients have pathogenic variants in the *MKS1*, *TMEM216*, and *TMEM67* genes (27), though more than 14 loci have been identified. This syndrome is characterized by a classic combination of occipital encephalocele, large polycystic kidneys, hepatic fibrosis, and postaxial polydactyly (27). Both fetuses in our cohort had enlarged echogenic kidneys, but an otherwise incomplete phenotype with polydactyly in one and an occipital encephalocele in the other. One pregnancy was carried to term and resulted in perinatal demise, in part due to hypoplastic left heart syndrome, a known MKS extra-renal anomaly (28).

BBS is also an autosomal recessive multisystem ciliopathy caused by mutations in more than 22 genes, the most disease-associated genes being *BBS1*, *BBS10*, and *BBS2* (29). Renal manifestations range from enlarged, echogenic kidneys with reduced cortico-medullary differentiation to a variety of CAKUT phenotypes, including duplex, ectopic or horseshoe kidney, or renal agenesis. In addition to polydactyly, extra-renal manifestations include retinal degeneration, obesity, intellectual impairment, and hypogonadism (27), which are typically not expressed in infancy, causing delay in the median age of clinical diagnosis to 9 years (30).

It is interesting to consider our single center data in the context of a recent report from Germany that analyzed 98 fetuses with bilateral polycystic kidney disease identified from the 20-year experience of referral centers in Bonn and Cologne (31). In this study, 90/98 (92%) fetuses had a genetically confirmed renal ciliopathy, whereas our study identified 72 fetuses with bilateral renal cystic disease from an 11-year experience, of which only 17/72 (24%) had a suspected renal ciliopathy, with genetic conformation in 13 (76%). The differences between these two contemporaneous studies are striking. Both were retrospective observational studies. In both cohorts, detailed fetal imaging and genetic evaluation were performed and the distribution of confirmed renal ciliopathies were comparable. It is possible that the total number of referred pregnancies in the German study were significantly higher than our study, given that the experience was twice as long and two large centers were involved. In addition, while the German study specifically focused on fetuses with isolated bilateral polycystic kidney disease, our study was more permissive, excluding CAKUT, chromosomal abnormalities, and syndromic disorders only in the final analysis of the suspected renal ciliopathy cohort.

Our study had as a second goal the systematic analysis of longitudinal post-natal follow-up for patients with bilateral renal cystic disease, including those with confirmed renal ciliopathies. Our analysis reveals that while 11/13 (85%) neonatal survivors with renal ciliopathies were referred for post-natal management to either our center's Inherited Renal Diseases Program or the General Nephrology clinic, nephrology care was much more limited for patients with bilateral renal cystic disease in the context of CAKUT (50%), chromosomal disorders (33%), or syndromes (17%). Only 2/6 patients with genetically-unresolved enlarged, echogenic kidneys at birth had documented post-natal nephrology follow-up.

Therefore, this single center study makes clear the need for a structure management plan as fetuses with renal cystic disease transition to post-natal life. Such a detailed framework for prenatal evaluation, delivery, and post-natal management of cystic renal disease was recently proposed by the European Network for Early Onset Cystic Kidney Disease (NEOCYST) consortium (32). Widespread implementation of these guidelines, similar to the model widely embraced for congenital heart disease (33, 34) would streamline and optimize the management of these neonatal survivors.

## Data Availability

The original contributions presented in the study are included in the article/Supplementary Material, further inquiries can be directed to the corresponding author.
